# Induced Obsessive Compulsive Symptoms in Patients with Psychosis Treated with Second Generation Antipsychotics: A Systematic Review

**DOI:** 10.1192/bjo.2026.11088

**Published:** 2026-06-30

**Authors:** Emma Leighton, Graham Walker, Filippo Queirazza

**Affiliations:** 1 University of Glasgow, Glasgow, United Kingdom; 2 NHS Greater Glasgow & Clyde, Glasgow, United Kingdom

## Abstract

**Aims:**

To identify quantitative studies of patients with psychotic disorders prescribed second generation antipsychotics (SGAPs), measuring new-onset or worsening obsessive compulsive symptoms (OCS) after treatment, and to evaluate whether SGAPs induce or exacerbate these symptoms.

**Methods:**

A systematic review was conducted using MEDLINE, EMBASE, CINAHL, PsycINFO and Web of Science, following PRISMA guidelines. Search terms covered psychosis, OCS and SGAPs. Of 2524 references retrieved, 1939 remained after duplicate removal. Screening by two independent reviewers identified 58 eligible abstracts, with 43 full-text studies included. The Yale–Brown Obsessive Compulsive Scale (Y-BOCS) was the most commonly used outcome measure.

**Results:**

Across 43 studies, 9044 participants with psychotic disorders were included, predominantly with schizophrenia (mean age 36 years). Clozapine was studied in 28 reports, with additional evidence for risperidone, olanzapine, ziprasidone and aripiprazole. Seventeen studies demonstrated statistically significant results, most associating clozapine with de novo OCS. Olanzapine was also linked to emergence or worsening of OCS symptoms, while adjunctive aripiprazole showed evidence of improvement in some cases. Most studies were observational, limiting causal inference.

**Conclusion:**

Preliminary evidence suggests clozapine is associated with new-onset OCS, potentially mediated by 5HT2a and 5HT2c antagonism. The association is less clear for other SGAPs, though there is evidence that olanzapine can contribute to a worsening in severity of OCS or emergence of new-onset OCS, and evidence that adjunctive aripiprazole can improve OCS outcomes. Further longitudinal, prospective trials are required to clarify these associations.

